# Pneumococcal Meningitis in a Region of Northern Spain, 1993–2023: Incidence Trends, Clinical Features, Recurrences, and Antibiotic Resistance

**DOI:** 10.3390/vaccines14020131

**Published:** 2026-01-28

**Authors:** Ayla Manzanal, Diego Vicente, Iñigo Ansa, Maitane Arrastia, Pedro Vallejo, José María Marimón

**Affiliations:** 1 Microbiology Department, University Hospital, Donostialdea Integrated Health Organization, 20014 Donostia-San Sebastian, Spain; 2 Department of Preventive Medicine, Faculty of Medicine, University of the Basque Country (UPV/EHU), 20014 Donostia-San Sebastian, Spain; 3 Microbiology Department, Infectious Diseases Area, Biogipuzkoa Health Research Institute, Donostialdea Integrated Health Organization, 20014 Donostia-San Sebastian, Spain

**Keywords:** pneumococcal meningitis, pneumococcal conjugate vaccines, antimicrobial resistance, recurrent meningitis

## Abstract

**Background:** *Streptococcus pneumoniae* is currently the leading cause of acute bacterial meningitis. This study assessed the impact of pneumococcal conjugate vaccines (PCVs) on pneumococcal meningitis in Gipuzkoa, north of Spain, between 1993 and 2023. **Methods:** All cases were serotyped and tested for antimicrobial susceptibility, with medical records reviewed since 2013. Overall, 193 patients were diagnosed (178 patients), averaging 6.2 cases annually. **Results:** Pneumococcal meningitis annual incidence decreased significantly after PCVs introduction, from 1.99 cases per 100,000 inhabitants in 1993–2001 (before PCV7) to 1.64 in 2002–2010 (PCV7 period) and further to 1.13 in 2011–2023 (PCV13 period). This decline was observed in all age groups except for adults aged ≥65 years, in whom the reduction was observed only after PCV13 introduction. The greatest reduction was observed in children under five. The incidence of meningitis caused by vaccine serotypes decreased following the progressive introduction of PCVs, but non-PCV serotypes increased from 0.70 to 0.95 cases per 100,000 between 1993–2001 and 2011–2023. Otitis media was the most common source of infection, followed by CSF fistula. Most cases (85%) required ICU admission; 67.5% showed sequels at discharge, mainly sensorineural hearing loss, and the 30-day mortality rate was 11.1%. Recurrent pneumococcal meningitis represented 7.8% of cases, associated with head trauma, with favorable outcomes and no mortality. Between 1993–2001 and 2002–2010, penicillin and cefotaxime resistance decreased from 25.4% to 13.3% (47.6% reduction) and from 19.7% to 5% (74.6% reduction), respectively. In 2011–2023, cefotaxime resistance stabilized, but penicillin resistance rose to 32.3%, mainly due to non-PCV13 serotypes. **Conclusions:** The use of PCV reduced the incidence of pneumococcal meningitis in the region, but penicillin resistance has increased in recent years, due to the rise in non-PCV13 serotypes.

## 1. Introduction

The epidemiology of acute bacterial meningitis (ABM) has undergone significant changes in the last three decades due to the introduction of conjugate vaccines against the three most common causative microorganisms: *Streptococcus pneumoniae*, *Haemophilus influenzae* type b, and *Neisseria meningitidis*. Their implementation in childhood vaccination schedules has led to a decrease in the overall incidence of ABM, demonstrating their efficacy and the herd protection they provide [1,2,3]. However, the effectiveness of pneumococcal conjugate vaccines (PCVs) has been partially limited by the emergence of non-PCV serotypes. Consequently, *S. pneumoniae* has become the leading cause of ABM [2,3].

Pneumococcal meningitis (PM) is a severe infection associated with high morbidity and mortality rates, and its clinical presentation is indistinguishable from ABM caused by other bacteria. The infection can arise from direct spread of the microorganism following otitis media or sinusitis, or through haematogenous dissemination [4]. Related to direct spread, *S. pneumoniae* is the most common cause of recurrent bacterial meningitis following head trauma, cerebrospinal fluid (CSF) fistulas, or cochlear implants [5,6]. Despite advances in healthcare, the mortality rate of PM in developed countries remains high (10–37%), and long-term sequelae are frequent [7,8]. Early initiation of appropriate treatment is the most critical factor influencing survival and the likelihood of complications.

In the pre-vaccine era, certain countries, like Spain, reported high rates of *S. pneumoniae* resistance to penicillin and third-generation cephalosporins [9,10]. PCVs significantly reduced antibiotic resistance by protecting against resistant serotypes. In recent years, there has been a reemergence of antibiotic-resistant serotypes not covered by PCVs [11,12].

In Gipuzkoa, PCV7 was introduced in 2001 on an unsubsidized basis with a progressive childhood coverage of 40–60%. This vaccine was replaced in 2010 by PCV13, which became part of the Public Health Service’s childhood vaccination schedule (subsidized) in 2015 and achieved over 95% coverage. The adult vaccination schedule includes the 23-valent pneumococcal polysaccharide vaccine (PPV23) for adults aged ≥65 years and younger adults belonging to risk groups. Vaccination coverage in the elderly remained below 25%.

The objective of this study was to analyze the incidence and clinical characteristics of PM in Gipuzkoa over the last three decades, the evolution of responsible serotypes, and the corresponding antibiotic susceptibility. Furthermore, cases of recurrent PM were investigated in detail to describe their clinical presentations, predisposing factors, disease course, and curative treatments.

## 2. Materials and Methods

The study included all PM cases diagnosed between 1993 and 2023 at the Donostia University Hospital (DUH), which has approximately 1000 beds and is located in Donostia-San Sebastián, Basque Country, Northern Spain. The population served by DUH has increased over the 31 years of the study, from 395,134 inhabitants in 1993 to 427,416 in 2023. This population represented 59% of Gipuzkoa’s total population in 2023 (720,529 inhabitants).

### 2.1. Case Definition

PM was defined as any case with a clinical diagnosis of ABM (mainly fever, headache, neck stiffness and altered mental status) and a compatible CSF analysis: pleocytosis (≥5 WBC/µL), neutrophil predominance (>80%), low glucose levels (<40 mg/dL), in which *S. pneumoniae* was isolated from CSF or from blood if not cultured from CSF. PM cases diagnosed by other microbiological methods, such as antigen or DNA detection in CSF, were excluded, as these techniques were not available throughout the whole study period (10 cases between 2010 and 2023).

### 2.2. Microbiological Techniques

*S. pneumoniae* was identified using the optochin susceptibility test and the bile solubility test throughout the study. Serotyping was performed using the Quellung reaction between 1993 and 2009, multiplex PCR between 2010 and 2017 [13], and S. Pneumostrip test (Operon, Zaragoza, Spain) [14], since 2017. Molecular tests were confirmed with the Quellung reaction. Serotypes were grouped as follows:

- PCV7: serotypes 4, 6B, 9V, 14, 18C, 19F, and 23F;
- PCV13: serotypes 1, 3, 4, 5, 6A, 6B, 7F, 9V, 14, 18C, 19F, 19A, 23F;
- PCV13-AS (the 6 additional serotypes of PCV13): serotypes 1, 3, 5, 6A, 7F, and 19A;
- Non-PCV13: serotypes not included in PCV13.

The antimicrobial susceptibility testing was performed using the broth microdilution technique (Sensititre, Thermo Scientific, The Netherlands). To avoid differences in interpretation due to changes in clinical breakpoints over time, all minimum inhibitory concentrations (MICs) were interpreted using the EUCAST 2025 clinical breakpoints [15]. The antimicrobials included in this study were penicillin, cefotaxime, and vancomycin.

### 2.3. Epidemiological and Clinical Data

Epidemiological variables (age, sex) were registered for the whole study period (1993–2023). Clinical data for all PM episodes were obtained from digitized medical records since 2013, the year in which complete digitized reports became available. The variables analyzed included: source of infection, need for Intensive Care Unit (ICU) admission, ICU length of stay (days), treatment, 30-day mortality, sequelae, and recurrences.

Recurrent PM (RPM) cases diagnosed during the entire study period (1993–2023) were selected, and their clinical data were reviewed.

### 2.4. Statistical Analysis

All incidence data were expressed as cases per 100,000 population/year. Trends in incidence were analyzed using Poisson regression models (SPSS v. 8.0).

To evaluate the effect of PCV7 and PCV13 in our region, the study was divided into three periods, and the incidence rates between them were compared using the rate ratio (RR) and the Poisson exact test (*p*-value):

- First or pre-PCV7 period (before PCV7 introduction): 1993–2001;
- Second or PCV7 period (after PCV7introduction): 2002–2010;
- Third or PCV13 period (after PCV13 introduction): 2011–2023.

The antibiotic resistance rates were also analyzed using the chi-square test or Fisher’s exact test when appropriate. A *p*-value of <0.05 was considered statistically significant.

## 3. Results

Between 1993 and 2023, 193 PM were diagnosed, corresponding to 178 patients. The annual average of PM was 6.2 cases (range: 0 to 13). The only year without any PM cases was 2020, coinciding with the onset of the COVID-19 pandemic. Of the 193 episodes, 120 (62.1%) occurred in males. The age distribution was as follows: 25 cases (13%) in children < 5 years old, 9 (4.6%) in children aged 5 to 14 years, 100 (51.8%) in adults aged 15 to 64 years, and 57 (29.5%) in adults ≥ 65 years old (in two cases, the age was unknown).

**Figure 1:**
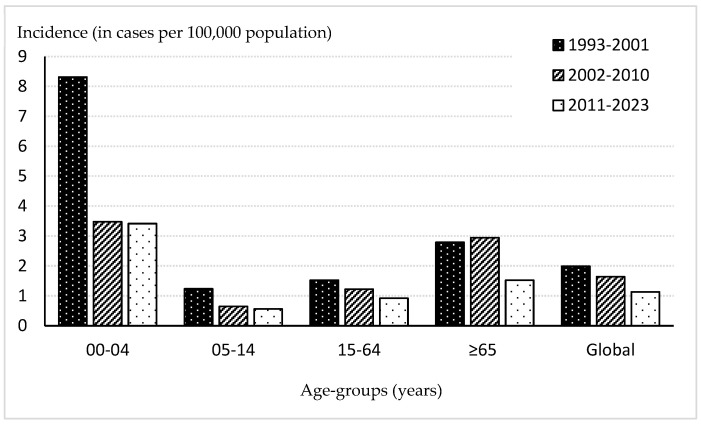
Average incidence of pneumococcal meningitis distributed by age groups, Gipuzkoa, Spain, 1993–2023.

The overall average incidence during the 31 years of study was 1.51 cases, highest in children < 5 years old with 4.4 cases, followed by adults ≥ 65 years old with 2.15 cases. Since the introduction of PCVs, there was a downward trend in the global average incidence (*p* < 0.01), decreasing from 1.99 in the pre-PCV7 (1993–2001) to 1.64 in the PCV7 period (2002–2010), and further declining to 1.13 after PCV13 implementation (2011–2023). This progressive decrease was observed across all age groups except for adults ≥ 65 years old (*p* = 0.59) (Figure 1).

The most significant reduction between the pre-PCV7 and PCV7 periods was observed in children < 5 years old, from an incidence of 8.31 to 3.48 (58.1% decrease). However, between the second and third periods (3.42 cases), no reduction was observed. In adults ≥ 65 years old, the incidence was maintained between the first and second periods, with incidences of 2.79 and 2.94, respectively (RR = 1.05; IC95%: 0.55–1.99; *p* > 0.05), decreasing to 1.64 in the third period (RR = 0.52, IC95%: 0.28–0.97, *p* = 0.047).

### 3.1. Serotype Distribution

The age group between 5 and 14 years was excluded from the serotype incidence analysis because there were only 9 cases of PM in the entire study, making it non-representative.

**Figure 2:**
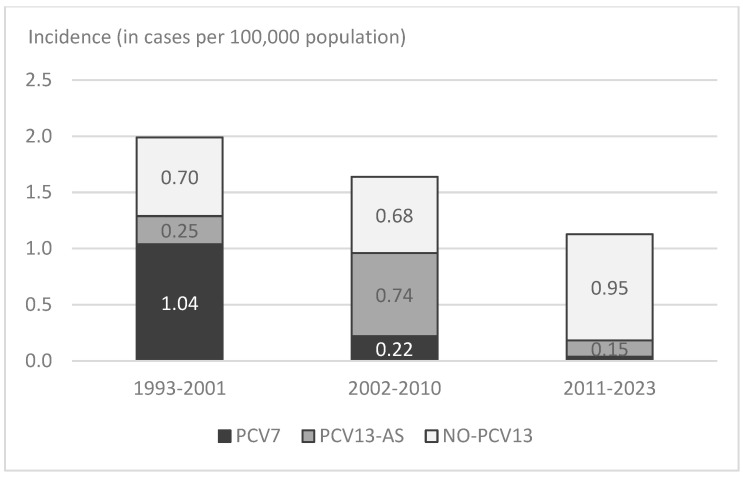
Average incidence of PM distributed by PCV serotypes, Gipuzkoa, Spain, 1993–2023.

The incidence of PM caused by PCV7 serotypes progressively decreased since its introduction (*p* < 0.001), from 1.04 to 0.22 and 0.04 over the three periods (Figure 2). Although the decrease occurred in all age groups, the greatest reduction was observed in children < 5 years, in whom the incidence of PM caused by PCV7 serotypes decreased from 7.56 to 0.58 and to 0.43 throughout the three periods.

Contrarily, the incidence of PM caused by PCV13-AS increased from 0.25 to 0.79 between the pre-PCV7 and PCV7 periods (RR: 3.145, IC 95%: 1.489–6.643, *p* < 0.001). This increase occurred in all age groups, mainly due to an increase in PM cases caused by serotypes 3 and 19A, which were involved in 33% (20/60) of all PM in 2002–2010.

After PCV13 introduction, the incidence of PCV13-AS significantly decreased to 0.15 (RR: 0.183, IC 95%: 0.084–0.401, *p* < 0.001). The largest reduction was observed in children < 5 years old, who had no PM caused by PCV13-AS after PCV13 implementation. Despite the decrease in all PCV13 serotypes, serotype 3 remained the most frequent among them (5/8).

The global incidence of PM caused by non-PCV13 serotypes exhibited an increasing trend over the study period (*p* < 0.001). The incidence remained stable between the pre-PCV7 (0.70 cases) and PCV7 (0.63 cases) periods (RR: 0.898, 95% IC: 0.51–1.58, *p* = 0.774), increasing to 0.95 in the PCV13 period (RR: 1.504, 95% IC: 0.920–2.456, *p* = 0.12). The largest increase was observed among children < 5 years, in whom the incidence of non-PCV13 serotypes increased from 0.76 to 1.74 and to 2.99 over the three periods. The most frequent non-PCV13 serotypes during the last period (2011–2023) were serotype 8 (6 cases), 23B (6 cases), 11A (5 cases), and 6C (5 cases).

### 3.2. Antimicrobial Susceptibility

**Figure 3:**
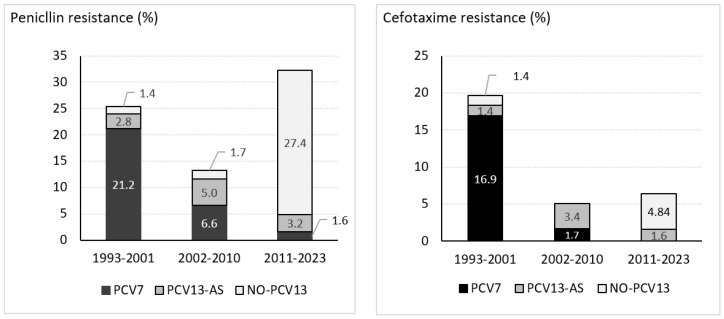
Penicillin and cefotaxime resistance rates of *S. pneumoniae* causing meningitis in Gipuzkoa, north Spain, 1993–2023.

Antimicrobial susceptibility was determined in all 193 isolates. Penicillin resistance (MIC > 0.06 mg/L) rate decreased from 25.4% (18/71) to 13.3% (8/60) between the pre-PCV7 and PCV7 periods (*p* < 0.05), mainly due to a decline in meningitis caused by penicillin-resistant PCV7 serotypes (Figure 3), especially serotype 14 (from 6 to 2) and 19F (from 4 to 1). In the PCV13 period, penicillin resistance increased to 32.3% (20/62) (*p* < 0.05), due to an increase in penicillin-resistant non-PCV13 serotypes, notably serotype 23B (6/20). Penicillin MIC50 remained <0.03 mg/L in the three periods, while MIC90 decreased from 2 mg/L in pre-PCV7 to 0.25 mg/L in PCV7 and PCV13 periods.

Cefotaxime resistance (MIC > 0.5 mg/L) rate decreased from 19.7% (14/71) in the pre-PCV7 period to 5% (3/60) in the PCV7 period (*p* < 0.05), due to a reduction in resistant PCV7-serotypes (from 12 to 1). After PCV13 implementation, cefotaxime resistance remained stable at 6.5% (4/62) (*p* = 0.7). Cefotaxime MIC50 was <0.06 mg/L over the three periods, while the MIC90 decreased from 1 mg/L to <0.06 mg/L and to 0.12 mg/L, respectively.

Since 2011, only four isolates exhibited cefotaxime resistance: two serotype 11A, one serotype 10A, and one serotype 19A. These four isolates were also penicillin-resistant with a MIC > 1 mg/L.

All *Streptococcus pneumoniae* isolates studied were vancomycin susceptible (MIC ≤ 2 mg/L).

### 3.3. Clinical Data

Between 2013 and 2023, 54 cases of PM were diagnosed in 52 patients (Table 1). Of these 54 cases, 68.5% occurred in men, and more than half of the patients were between 15 and 64 years old. The most common source of infection was otitis media, followed by CSF fistula. Eighty-five percent of cases required ICU admission, with an average stay of 5 days (range: 1–21 days). Sequelae at discharge were noted in 67.5% of cases, with sensorineural hearing loss being the most common. The 30-day mortality rate during the eleven-year period was 11.1%.

### 3.4. Recurrent Pneumococcal Meningitis (RPM)

Of the 193 episodes of PM diagnosed between 1993 and 2023, 15 (7.8%) were recurrent episodes, corresponding to 10 patients. Three of them had had some episodes of RPM prior to 1993. The mean number of RPM per patient was 2.5 cases (range 2–4 cases). Most (70%) were men, and the mean age at the first episode was 31 years (range 2–57 years). The time between the first and second episodes was on average 4 years (range 0.5–13.6 years). All recurrent episodes were caused by a different serotype (reinfections) (Table 2).

Regarding the predisposing factors of RPM, eight patients had a history of traumatic brain injury (TBI) with a basal skull fracture and subsequent communication with the paranasal sinuses. In the ninth patient, a CSF leak was found due to a congenital bone defect of the tegmen tympani. The clinical records of the 10th patient could not be found. All patients underwent surgery; three of them required more than one intervention until the leak was closed. After bone defect repair, no patient experienced a recurrence. All patients with RPM had a favorable outcome, and 30-day mortality was 0%.

## 4. Discussion

The overall incidence of PM in Gipuzkoa decreased (from 1.99 to 1.64 cases) after the implementation of PCV7 in the childhood vaccination schedule in 2001. This reduction, also observed in other Spanish studies [16], occurred at the expense of a 79% decrease in PM caused by PCV7 serotypes. The US Active Bacterial Core Surveillance showed a decrease in PM incidence from 1.09 to 0.81 between 1998–1999 and 2006–2007, due to a 92% reduction in PCV7 serotypes [17]. European studies conducted in France, Sweden, the Netherlands, the UK, and Spain also showed decreases in PCV7 serotypes [8,16,18,19,20], although the effect on the incidence of PM varied among countries.

In France, due to the high degree of non-PCV7 serotype replacement (mainly PCV13-AS), a significant increase in the number of PM of 22% and 18% was observed in 2008–2009 and 2009–2010, respectively, compared to the baseline period of 2001–2003, thus voiding the effect of PCV7 [18]. In the UK, in children < 5 years, the incidence of PM decreased despite serotype replacement [21]. However, in adults who had a low proportion of PCV7 serotypes prior to PCV7 introduction, the emergence of non-PCV7 serotypes led to a stable incidence of PM.

After PCV7 introduction in Gipuzkoa, the incidence of PCV13-AS increased from 0.25 to 0.79 cases. As reported in the USA and several European countries, including Spain, the multidrug-resistant serotype 19A was especially involved in the increase in PCV13-AS [22,23]. The incidence of PM caused by serotype 3 also increased after PCV7 introduction, as observed in IPD in Sweden [19], and specifically in PM in England, where serotype 3 was one of the main causative serotypes after the use of PCV7 [20].

In Gipuzkoa, PCV13 was implemented in 2010, successfully reducing the overall incidence of PM to 1.13 cases in 2011–2023, despite only children < 1 year of age being vaccinated. As happened after PCV7 introduction, the replacement with non-PCV13 serotypes (a 40% increase) did not avoid the progressive decline in the incidence of PM.

Since 2010, most countries, including Spain, substituted PCV7 with PCV13. However, some other countries, such as Lithuania, Austria, Bulgaria, the Netherlands, Serbia, and Belgium, decided to implement PCV10 (Belgium and Serbia initially introduced PCV13, but switched to PCV10 for economic reasons) [24,25]. In the Netherlands, data collected up to 2014 showed a reduction in the overall incidence of PM, with no observed replacement by non-PCV10 serotypes [8]. In Belgium, contrarily, two years after switching to PCV10, a significant increase in IPD caused by serotype 19A (a serotype covered by PCV13 but not by PCV10) was observed [24]. In other countries, such as Brazil [26] and Mozambique [27], with high coverage rates over 90%, the introduction of PCV10 achieved important declines in pediatric PM (56.5% and 94.3%, respectively). In Japan [28], the introduction of PCV13 in routine pediatric vaccination was not accompanied by a reduction in adult PM. In these countries, a serotype replacement by non-vaccine-covered serotypes was also observed.

In France and the UK, where PCV7 failed to reduce the incidence of PM, PCV13 succeeded. In the UK, a 48% reduction in PM was achieved in 2015–2016, due to the decrease in PCV13 serotypes, without replacement by non-PCV13 serotypes [29]. In France, in the four years following the PCV13 implementation, a decrease in PM incidence was reported for the first time, also without an increase in non-PCV13 serotypes [18]. However, two years later, in 2016, a nationwide prospective pediatric study found an increase in PM caused by non-PCV13 serotypes, bringing the incidence back to previous levels [30]. This later study raises the question of whether the increase in PM caused by non-PCV13 serotypes is a matter of time. The lack of recent studies on the epidemiology of PM makes it difficult to clarify this question, although our data suggest that, as in France, non-PCV13 serotypes are replacing those disappearing after PCV13 introduction.

Prior to the introduction of PCVs, *S. pneumoniae* exhibited high rates of resistance worldwide [31,32,33,34]. In our region, the penicillin and cefotaxime resistance rates in 1993–2001 were 25.4% and 19.7%, respectively, similar to those from IPD studies conducted in Spain [32,35]. The high rates of beta-lactam resistance led the IDSA and ESCMID to modify the empirical treatment guidelines of PM, recommending adding vancomycin to the treatment regimen, at least until the antimicrobial susceptibility results became available [36,37].

In the pre-PCV7 period, over 80% of penicillin and cefotaxime-resistant isolates were PCV7 serotypes. Consequently, after PCV7 implementation, significant decreases of 47.6% and 74.6% in penicillin and cefotaxime resistance, respectively, were observed. The US Active Bacterial Core Surveillance also assessed a reduction in the resistance of *S. pneumoniae* causing IPD and meningitis following PCV7 introduction [38,39]. Specifically for PM, a progressive decline in the incidence of penicillin and cefotaxime-resistant isolates between 1998–1999 and 2004–2005 was reported [38].

However, PCV13 did not achieve the same effect, since penicillin resistance increased to 32.3% and cefotaxime resistance remained at 6.5%. The increase in penicillin resistance was caused by non-PCV13 penicillin-resistant serotypes, especially due to serotype 23B, which has demonstrated CNS tropism [40]. The six penicillin-resistant strains corresponding to serotype 23B exhibited a MIC of 0.25 mg/L. This finding aligns with reports from Switzerland, where a recent increase in IPD due to serotype 23B was observed after the COVID-19 pandemic, with these isolates showing penicillin MICs between 0.064 and 0.19 mg/L (resistant according to meningitis breakpoints) [41].

After PCV13 introduction, only three non-PCV13 isolates of serotypes 10A and 11A (*n* = 2) showed high-level resistance to penicillin and cefotaxime (≥1 µg/mL). Serotype 11A has emerged as a serotype of concern, due to its association with penicillin resistance, its high prevalence in IPD [42,43]. In Spain, serotype 11A strains already presented a MIC90 of 2 mg/L in 2010–2011, a figure that increased to 4 mg/L after the COVID-19 pandemic [12,44]. However, the remaining non-PCV13 penicillin-resistant serotypes (*n* = 17) showed low-level resistance to penicillin (MICs 0.12–0.25 mg/L) and remained susceptible to cefotaxime. The emergence of non-PCV13 serotypes with low-level penicillin resistance has also been observed since 2014 in Spain [43].

Despite the increase in penicillin resistance in the post-PCV13 period, the rate of cefotaxime resistance remained low, making the use of only third-generation cephalosporins adequate for the empirical treatment of PM in our region. Although there are few recent studies reporting data on resistance rates in PM, a 2016 study in Israel also proposed third-generation cephalosporins as empirical treatment due to the absence of cefotaxime resistance [33].

In the last three decades, PM mortality has decreased significantly thanks to early treatment and PCV use. However, the morbidity and mortality associated with this infection remain high today [8]. In Gipuzkoa, in the last decade, 85% of PM required ICU admission, 16.7% presented sequelae upon discharge, and the mortality rate was 11.1%, similar to data reported from other developed countries, confirming the severity of this infection [1,2,45].

One of the most common PM sequelae is hearing loss, which affects 7–36% of patients [45,46,47]. Otitis media, as the source of the infection, is the main risk factor for developing hearing loss after PM [47,48]. In Gipuzkoa, 9.3% (5/54 cases) of PM diagnosed since 2013 presented sensorineural hearing loss, a percentage that rises to 27.8% (4/18) in PM with an otic focus. According to a Dutch study, another risk factor is the presence of serotype 3 [47]. In this study, no predominant serotype was observed among the cases of hearing loss (all cases caused by different serotypes).

Cerebrovascular accidents (CVAs), although less common than hearing loss, are of particular importance in PM, as they occur more frequently than in other bacterial meningitides and cause significant sequelae. Neurological deficits resulting from CVAs may be transient or persistent upon discharge, interfering with patients’ quality of life in the latter case [2,49]. In this study, two patients showed sequelae at discharge derived from stroke: one diplopia due to paresis of the VI cranial nerve and left hemiparesis due to ischemic stroke.

Recurrent bacterial meningitis accounts for 5–7% of ABM cases, and unlike acute meningitis, its prognosis is relatively favorable, with significantly lower mortality rates. The most frequently implicated microorganism is *S. pneumoniae*, probably due to its presence in the normal nasopharyngeal microbiota [50,51]. The most important predisposing factors are head trauma, congenital malformations, and immunosuppression. In the case of RPM, the most common predisposing factor is head trauma with a skull base fracture [5,50].

In Gipuzkoa, all patients with RPM, whose medical history was obtained, had predisposing factors, most notably head trauma, present in 80% of cases. Men represented 70% of the patients, a percentage similar to that reported by the Dutch Meningitis Cohort Study in 2007, and which seems to be attributable to the fact that TBI is more common in men [51]. In all cases, recurrences ceased when the bone defect was repaired, and the CSF fistula closed. Therefore, in a patient with RPM, the predisposing factor must be identified and, if it is a fracture or bone malformation, it must be repaired, since this is the definitive treatment for recurrences.

Our study was carried out in a relatively small region, which may limit the generalizability of the findings to larger populations. However, conducting the study within the same center and using consistent diagnostic procedures over time ensured the homogeneity of the collected data throughout the 31-year study period.

## 5. Conclusions

This study highlights the efficacy conferred by PCVs with a progressive decrease in the incidence of PM since their introduction. Furthermore, it underscores the challenge posed by serotype replacement, which impacts not only the incidence but also the rate of antibiotic resistance. Therefore, given that it is a severe infection that can lead to significant sequelae, it is crucial to improve elderly vaccination coverage and continue monitoring IPD epidemiology and, consequently, that of meningitis.

## Figures and Tables

**Table 1:** Clinical and demographic data of PM diagnosed between 2013 and 2023.

	n	(%)
Gender - Women - Men	17 37	(31.5) (68.5)
Age - <5 years - 5–14 years - 15–64 years - ≥65 years	6 2 29 17	(11.1) (3.7) (53.7) (31.5)
Source of infection - Otitis media - Sinusitis - CSF fistula - Middle ear ^1^ - Paranasal sinuses - Pneumonia - Unknown	18 5 12 7 5 4 15	(33.3) (9.2) (22.2) (7.4) (27.8)
ICU admission	44	(85)
Sequel at discharge - Sensorineural hearing loss - Vertigo (vestibular nerve damage) - Diplopia (cranial nerve 6 paresis) - Ischemic stroke (left hemiparesis) - Vasculitis	9 5 1 1 1 1	(16.7) (9.3) (1.8) (1.8) (1.8) (1.8)
30-day mortality - <15 years - ≥15 years	6 1 5	(11.1) (1.9) (9.2)

^1^ Three of the CSF fistulas with communication with the middle ear corresponded to the same patient.

**Table 2:** Recurrent pneumococcal meningitis among 193 cases of PM, Gipuzkoa, Spain, 1993–2003.

Patient	Gender	Age at 1st Episode (Years)	Number of RPM	Episode Date	Episode Serotype
**1**	F	35	4 * 5 episodes before 1993	1st 06/1994 2nd 09/2000 3rd 03/2006 4th 01/2007	11A 18C 3 23F
**2**	M	33	2 * 1 episode before 1993	1st 12/1996 2nd 04/2001	23F 4
**3**	F	45	3 * 1 episode before 1993	1st 04/1995 2nd 12/2001 3rd 11/2002	15B 4 16F
**4**	M	26	2	1st 04/1993 2nd 06/1995	6A 19F
5 *	M	2	2	1st 04/1994 2nd 12/1997	6B 14
**6**	M	32	2	1st 05/1996 2nd 08/1997	9V 6B
**7**	F	5	3	1st 05/1997 2nd 12/1997 3rd 10/1998	11A 35 38
**8**	M	38	2	1st 05/2003 2nd 02/2006	14 4
**9**	M	57	2	1st 04/2005 2nd 12/2018	31 11A
**10**	M	38	3	1st 12/2021 2nd 01/2023 3rd 06/2023	6C 6D 8

* No clinical records were found.

## Data Availability

The original contributions presented in this study are included in the article. Further inquiries can be directed to the corresponding author.

## Abbreviations

The following abbreviations are used in this manuscript:

ABM	Acute bacterial meningitis
CVA	Cerebrovascular accident
ICU	Intensive Care Unit
IPD	Invasive pneumococcal disease
MIC	Minimum inhibitory concentrations
RPM	Recurrent pneumococcal meningitis
PCV	Pneumococcal conjugate vaccine
PM	Pneumococcal meningitis
PPV	Pneumococcal polysaccharide vaccine
TBI	Traumatic brain injury
